# Preliminary laboratory analytical comparison of quantitative at-home urinary hormone monitoring systems using artificial urine

**DOI:** 10.3389/fendo.2026.1947016

**Published:** 2026-10-19

**Authors:** Thomas P. Bouchard, Paul J. Yong, Patricia K. Doyle-Baker

**Affiliations:** 1 Department of Family Medicine, University of Calgary, Calgary, AB, Canada; 2 Department of Obstetrics & Gynecology, University of British Columbia, Vancouver, BC, Canada; 3 Faculty of Kinesiology, University of Calgary, Calgary, AB, Canada

**Keywords:** analytical performance, device validation, estrone-3-glucuronide, fertility monitor, follicle-stimulating hormone, luteinizing hormone, pregnanediol glucuronide, specificity

## Abstract

**Background:**

Quantitative at-home urinary hormone monitoring systems are increasingly used for reproductive health tracking, but their analytical behaviour requires systematic characterization. This preliminary laboratory comparison aimed to provide an analytical framework to compare multiple commercially available at-home urine hormone monitoring devices across key performance metrics under controlled artificial urine conditions.

**Methods:**

Laboratory experiments were informed by Clinical and Laboratory Standards Institute’s (CLSI) validation principles. Four urine hormone monitoring devices were evaluated using a pre-registered analytical framework for luteinizing hormone (LH), follicle-stimulating hormone (FSH), estrone-3-glucuronide (E3G), and pregnanediol glucuronide (PDG). Metrics included coefficients of variation (CV, for precision), relative deviation (RD, for accuracy), linearity, limit of blank (LoB), limit of detection (LoD) for each device, using artificial urine samples with standard hormone concentrations. Cross-reactivity was tested with other hormones including human chorionic gonadotropin (hCG), cortisol, thyroid stimulating hormone (TSH) and progesterone subtypes. Device-specific evaluations, including the impact of ambient light and various camera systems, were also conducted for relevant devices.

**Results:**

There were device- and analyte-specific differences in precision, accuracy, linearity and detection limits. Precision and accuracy ranged from CV/RD values near the 15% acceptability threshold to levels exceeding 50% at some concentrations. External lighting conditions affected camera-based strips at low hormone concentrations. False positive results for LH can occur in the presence of 100 mIU/mL hCG, which could lead to misinterpretation in the context of pregnancy. One device that measures the LH-beta subunit avoided this cross-reactivity.

**Conclusions:**

A preliminary analytical framework was applied to evaluate the performance of quantitative urinary hormone devices for at-home use. This framework can be used in future research and development of menstrual health monitoring technologies. Longitudinal clinical studies using human urine samples and appropriate ovulation reference standards are required before conclusions can be drawn regarding ovulation detection, fertile-window estimation or menstrual-phase classification.

## 1 Introduction

The ability to accurately monitor reproductive hormones at home has become increasingly important for individuals seeking to improve menstrual health literacy (1) and manage their fertility (2, 3). In addition to personal menstrual health knowledge, the clinical value of prospective hormone tracking facilitates clinical decision-making: (i) identifying whether a cycle is ovulatory or anovulatory (4–6), (ii) narrowing the fertile window to support appropriately timed intercourse (7, 8), (iii) documenting cycle phase patterns that may prompt evaluation of ovulatory dysfunction (9, 10), and (iv) supporting longitudinal monitoring in populations where cycle variability is common, e.g., postpartum, perimenopause, endocrine disorders (11) and female athletes at risk of menstrual dysfunction (12). Nevertheless, devices need to be evaluated to ensure they are accurate and precise in their ability to assist decisions to continue timed attempts to conceive (7, 13), initiate or adjust ovulation induction (13), or escalate to more in-depth clinical evaluation (14).

Fertility monitoring has historically relied on self-assessment of symptoms or clinical assessment with ultrasound and serum-based hormone panels (15). Given barriers to access (16) and difficulty accessing a clinician in resource-constrained environments (17), there is a demand for user-friendly at-home solutions, empowering individuals to take an active role in managing their reproductive health (3, 18). The four devices evaluated in this study providing real-time data on hormonal fluctuations throughout the menstrual cycle (19), measuring four key reproductive hormones: luteinizing hormone (LH), follicle stimulating hormone (FSH), urine estrogen metabolites (estrone-3-glucuronide, E_1_3G), and urine progesterone metabolites (pregnanediol glucuronide, PDG). The pituitary hormones, LH and FSH, are large glycoproteins excreted in urine intact. The two ovarian steroid hormones (estradiol and progesterone) undergo modification in the liver before excretion. Estradiol (E2) is oxidized into the weaker estrone (E1) and that is then glucuronidated into water soluble E_1_3G for urinary excretion. Progesterone is reduced into pregnanediol and then glucuronidated into water soluble PDG for urinary excretion.

Monitoring these hormones can facilitate estimation of the fertile window for achieving and avoiding pregnancy and for monitoring perimenopausal status (11). This requires knowing whether each device is analytically accurate enough to monitor hormonal physiology. This includes identifying whether there is any cross-reactivity with other hormones that could be present in the urine (20) with implications for monitoring exogenous hormones as in hormone replacement therapy (21, 22). Synthetic progestins and estrogens are structurally distinct and not measured by these devices. Oral bioidentical estradiol and progesterone would disproportionally elevate urinary E_1_3G and PDG because first-pass metabolism floods the liver with substrate, compared to endogenous hormones. Transdermal hormones, which largely bypass the liver, would be underestimated by these devices. These exogenous hormone caveats need to be clearly understood in the context of widespread use of these devices when hormonal medications are used, or other hormonally disrupting medications are used (e.g. chemotherapy).

Of the four devices studied here, there are previous published studies on serum-urine correlations for the Device 1 (23), ultrasound and serum validation for Device 2 (24), and urinary menstrual cycle profiling for Device 3 and Device 4 testing systems (10, 25). Device 2 has now been validated with ultrasound-confirmed ovulation as well as serum hormones (26). Analytical limitations in each device or cross-reactivity with structurally related hormones (27) may lead to misinterpretation, such as falsely suggesting a fertile window, missing an LH surge, incorrectly “confirming” ovulation, or generating confusing signals that increase anxiety and delay appropriate care. Rigorous analytical validation is therefore a necessary precursor to clinical claims and to the responsible integration of at-home monitoring into fertility care pathways (28).

In this study, the primary aim was to define an analytical evaluation framework and demonstrate its use by applying it to four commercially available at-home urinary hormone monitors. We analyzed a dataset that directly compares analytical performance of commercially available at-home monitors under controlled laboratory conditions with a pre-registered publicly available analysis plan (29).

## 2 Materials and methods

### 2.1 Setting

We analyzed a dataset provided by one of the monitor manufacturers (Quanovate Tech Inc., San Diego, CA, USA) after they performed testing of four devices in their facilities. Operational procedures included recording laboratory environmental conditions and device serial numbers for all tests. All testing was conducted following the manufacturer’s instructions for each device and photos were taken of each testing scenario. Artificial urine was utilized for preparing a standard (“spiked”) solution of hormones at specified concentrations. Commercial hormone standards were obtained for LH (catalogue no. 200531, Scripps Laboratories, San Diego, CA, USA), FSH (catalogue no. 2754401, Scripps Laboratories, San Diego, CA, USA), E_1_3G (catalogue no. 0000279679, Sigma-Aldrich, St. Louis, MO, USA), and PDG (catalogue no. 0000400053, Sigma-Aldrich, St. Louis, MO, USA). Standards were diluted in artificial urine (catalogue no. 2503130010, Beijing Solarbio Science & Technology Co., Ltd., Beijing, China). Detailed documentation of sample preparation was maintained, including pH and specific gravity checks to ensure matrix consistency.

### 2.2 Monitors tested

Device 1 was tested measuring all four hormones (FSH, E_1_3G, LH and PDG). Device 2 had two different wands that were tested (Device 2a measuring E_1_3G, LH and PDG and Device 2b measuring all four hormones). The Device 3 and Device 4 testing systems measured E_1_3G, LH and PDG (Device 4 uniquely measures the beta subunit of LH). Both Device 3 and Device 4 systems can now measure FSH, but this functionality was not available at the time of testing for Device 3 and there was no artificial urine FSH data for Device 4. To minimize commercial bias, we have coded the devices in alphabetical order in Figures and Results as Device 1, Device 2a, Device 2b, Device 3, and Device 4. The specifics of each device are identified in the online analysis plan (29). **Table 1** summarizes the features for each device. One other urine hormone device was analyzed, but because it did not provide quantitative hormone data, it was not included in this analysis; otherwise, all pre-specified measurements were included in the analysis.

**Table 1:** Comparison of analytical performance characteristics across the five evaluated urinary hormone monitoring device configurations.

Feature	Device 1	Device 2a	Device 2b	Device 3	Device 4
Format/hardware	Reader-based, controlled lighting	Reader-based, controlled lighting	Reader-based, controlled lighting	Strip-based, smartphone imaging	Strip-based, smartphone imaging
Hormones measured	LH, FSH, E_1_3G, PDG	LH, E_1_3G, PDG	LH, FSH, E_1_3G, PDG	LH, E_1_3G, PDG	LH (β-subunit), E_1_3G, PDG
Precision and accuracy for LH/FSH	Acceptable at some levels; often >15% CV/RD at others	Near or below 15% across most concentrations	Near or below 15% across most concentrations	Frequently >15% CV/RD	Frequently >15% CV/RD
E_1_3G performance	High precision, substantial systematic bias (poor accuracy)	High precision and accuracy across all concentrations	Good precision and accuracy, slightly lower than 2a	Poor precision and accuracy	Poor precision and accuracy
PDG performance	Best overall accuracy; strong linearity	Good accuracy and sensitivity, especially at higher concentrations	Adequate linearity; higher LoD	Poor linearity and accuracy	Poor linearity and accuracy
LoB/LoD (overall)	Strong for LH/FSH and PDG; good for E_1_3G	Good for E_1_3G and PDG; LoD near clinical thresholds	Good for LH/FSH; moderate for E_1_3G	Higher LoD, especially for E_1_3G/PDG	Highest LoD; weaker low-end performance
hCG cross-reactivity on LH	Marked LH signal at 100 mIU/mL hCG	Low-to-moderate LH signal at 100 mIU/mL hCG	Low-to-moderate LH signal at 100 mIU/mL hCG	Highest LH signal at 100 mIU/mL hCG	No detectable LH signal at 100 mIU/mL hCG (β-specific)
Cross-reactivity with other hormones	Some cross-reactivity but clinically negligible	Some cross-reactivity but clinically negligible	Some cross-reactivity but clinically negligible	Some cross-reactivity but clinically negligible	Some cross-reactivity but clinically negligible
Ambient light sensitivity	None (controlled illumination)	None (controlled illumination)	None (controlled illumination)	Modest effects at some low concentrations	More pronounced effects, especially at low LH and PDG

CV, coefficient of variation; E_1_3G, estrone-3-glucuronide; FSH, follicle-stimulating hormone; LH, luteinizing hormone; PDG, pregnanediol glucuronide; hCG, human chorionic gonadotropin; LoB, limit of blank; LoD, limit of detection; RD, relative deviation.

All testing was done over four days by five operators under conditions specified by each manufacturer. Device 1 assays were done using two instruments and three lots of test strips (the same lot was used for the replicates of 10). Device 2a/b assays were done using a single device with one lot of 2a, and one lot of 2b wands (same lot for replicates of 10). Each device and test strip combination had 208 test runs. All device and test strip serial numbers are included in the Appendix.

### 2.3 Performance validation tests

The following experiments were informed by validation principles based on the Clinical and Laboratory Standards Institute (CLSI, Wayne, PA, USA), with relevant standards referenced where applicable. The experiments did not constitute full CLSI-compliant validation but preliminary estimates as a framework. Full analytical validation would require additional design considerations to incorporate between-day, between-run, lot-to-lot and instrument-to-instrument comparisons.

#### 2.3.1 Precision and accuracy evaluation

Precision and accuracy were informed by CLSI standard EP05-A3 (30). Three concentration levels spanning different ranges for each hormone were tested 10 times per device and hormone concentration (LH and FSH: 12.5, 25, 50 mIU/mL; E_1_3G: 75, 150, 300 ng/mL; PDG: 2.5, 5, 10 μg/mL). Coefficients of variation (CV) were computed as (standard deviation/mean × 100) to quantify device precision, and relative deviation (RD) was computed as (measured – expected)/expected × 100 to quantify accuracy as the absolute proportional difference between measured and reference concentrations. CV values quantify the magnitude of random measurement error at each concentration, while RD values quantify the magnitude of systematic bias from the true reference value. The International Council for Harmonization (ICH) guidelines on accuracy for bioanalytical assays specifies that precision and accuracy should not exceed ±15% of each tested concentration level, and this was used as a benchmark for interpretation of results (31). For each device–analyte–concentration combination, CV and RD were calculated from the 10 replicate measurements, resulting in one summary value per experimental cell. Because these summary values were derived from technical replicates obtained under very similar experimental conditions, they were not treated as independent measurements of population-level device performance. The summary values were used in a mixed-effects model (device as fixed effect, concentration as random effect) with log-transformation. Compact letter displays and are exploratory and should not be interpreted as formal evidence of generalizable between-device differences.

#### 2.3.2 Linearity testing

Linearity was evaluated for each device-analyte combination by linear regression of measured versus expected concentrations across five concentration levels (10 replicates per level), with the coefficient of determination (R^2^) reported based on CLSI standard EP06 (30). Proportional bias in linearity was addressed by calculating the slope and intercept with 95% confidence intervals with deviation from linearity reported as maximum allowable deviation from linearity. Unlike the benchmarks for precision and accuracy, there is no specific acceptance threshold so descriptive statistics were used rather than statistical comparisons between devices. Since these were exploratory analyses, compact letter displays are meant for descriptive rather than inferential comparisons.

#### 2.3.3 Limit of Blank determination

The Limit of Blank (LoB) was determined based on the CLSI standard EP17-A2 (32) by testing analyte-free (concentration of 0) artificial urine 20 times for each device-analyte combination. For each replicate set, the mean and standard deviation (SD) of the recorded readings were computed, and the LoB was calculated as LoB = mean(blank) + 1.645×SD (blank). This statistic represents the highest reading expected to be observed when no analyte is present, allowing distinction between true negative measurements and background noise inherent to each device’s assay.

#### 2.3.4 Limit of Detection determination

The Limit of Detection (LoD) evaluation was based on the same CLSI standard as LoB (32) by testing standard samples spiked with low analyte concentrations (5 mIU/mL for LH and FSH, 40 ng/mL for E_1_3G, and 2.5 µg/mL for PDG) 20 times for each device-analyte combination. Building on the device-specific LoB, the LoD was calculated as LoD = LoB + 1.645×SD (low-concentration sample), incorporating both blank-sample noise and the additional measurement variability introduced at low analyte concentrations. This statistic represents the lowest analyte concentration that can be reliably distinguished from blank with 95% confidence.

#### 2.3.5 Lighting test for reagent strips

For reagent strips without stable light sources (Device 3, Device 4), ambient light tests were conducted under varying illumination conditions, with each concentration and condition repeated 10 times for three hormones (LH, E_1_3G, PDG). The three lighting conditions were regular ambient light (reference), 50 lux (dark), and 300 lux (bright).

#### 2.3.6 Specificity investigation

Specificity for each hormone was evaluated by testing test concentrations of hCG (100 mIU/mL), which shares a subunit with LH, as well as thyroid stimulating hormone (TSH, 0.025 mIU/mL), 17-^α^-hydroxyprogesterone (17^α^-OHP, 5 µg/mL), pregnenolone (5 µg/mL), cortisol (5 µg/mL), and progesterone (5 µg/mL). Each device-analyte-interferent combination was tested in triplicate to identify potential cross-reactivity.

### 2.4 Statistical analyses

Accuracy and precision (CV and RD) were compared between devices with ANOVA with *post-hoc* pairwise comparisons (significance level p<0.05, Tukey-adjusted) and were summarized using compact letter displays (CLDs) in heatmaps to identify significant device-level differences between devices. A similar statistical comparison with CLDs was also done for the specificity comparisons (analysis of interferent hormones) between devices.

Linearity was evaluated by regressing device-reported concentrations against standard concentrations to provide coefficients of determination (R^2^). For the lighting test, bright and dark conditions were compared to ambient light with a linear mixed-effects model with each test as a random effect and bright and dark conditions as fixed effects using Dunnett’s many-to-one test with multiplicity correction. All analyses were performed in R version 4.3.2 (R Core Team, 2024) with Positron (Posit Software, PBC, Boston, MA, 2024), using Claude (Anthropic AI) for code development without exposure to actual data. Specific R packages included lme4, emmeans, and multcomp, multcompView.

### 2.5 Ethical considerations

Given these tests were done on artificial urine spiked for specific hormone concentrations, no human volunteers were needed and therefore was exempt from ethics review. The analytic validation tests below were part of the pre-registered analysis plan (29) established to facilitate reproducibility and *post-hoc* analytical flexibility, however data collection occurred prior to the pre-registered data plan. Quanovate Tech Inc analyzed the data on their own but were not involved in the pre-registered data plan and did not see the results before publication. The manufacturer was not involved in the statistical analysis, interpretation nor manuscript preparation, these were carried out independently by the academic authors.

## 3 Results

An overview of each device and its performance is shown in **Table 1**, including strengths and limitations of each.

### 3.1 Analytical precision and accuracy

As shown in the **Figure 1** heatmap, for LH and FSH, Devise 2a and 2b consistently achieved CV precision levels at or near 15% threshold across all three concentrations, while the others exceeded this threshold for LH and FSH at most concentrations (though Device 1 at low concentration FSH was acceptable). Accuracy followed a similar pattern: Device 2a and 2b achieved RD near or below the 15% threshold at most concentrations, with the others showing substantial systematic bias, with RD values exceeding 50% at several LH concentrations.

**Figure 1:**
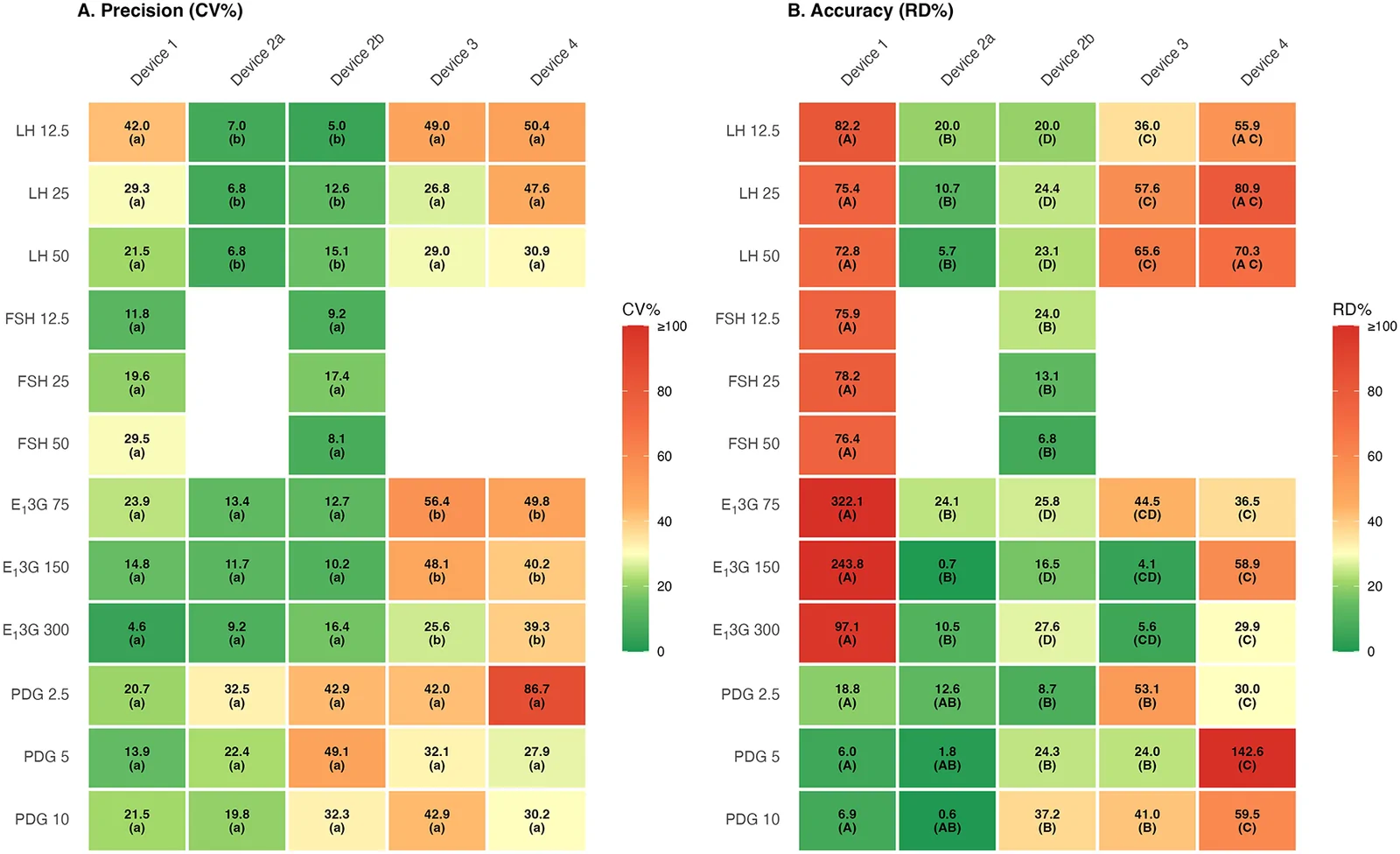
Heatmap of precision and accuracy for all devices relative to three concentrations of each hormone. The colour scale represents darker green values having the best precision (low CV%) and accuracy (low RD%) towards yellow, orange then red for worsening precision/accuracy. Letters in parentheses indicate compact letter displays from Tukey-adjusted pairwise comparisons within each analyte, but are shown for descriptive purposes rather than formal device-level inferential comparisons. A shared letter indicates a similar (not significantly different) pattern between devices. CV%, coefficient of variation; RD%, relative deviation; E_1_3G, estrone-3-glucuronide; FSH, follicle stimulating hormone; LH, luteinizing hormone; PDG, pregnanediol glucuronide. Where blank for FSH, this was not measured for this device. Data represent mean values from n=10 replicates per level in artificial urine.

For E_1_3G, Device 2a achieved acceptable precision and accuracy across all concentrations, while Devices 3 and 4 showed both elevated CV and RD across the tested range. Notably, Device 1 demonstrated low CV for E_1_3G, suggesting good precision, but markedly elevated RD% (97-322%), indicating systematically biased relative to spiked for PDG, Device 1 and 2a showed higher apparent concentrations and better alignment with target values than the others.

### 3.2 Linearity

**Figure 2** shows the heatmap for linearity coefficients of determination (R^2^) for all the hormones across the four devices, with **Table 2** showing proportional bias and non-linear patterns. For LH, all devices had high R^2^ values, but only device 2a had a slope close to 1 and low allowable deviation from linearity (8.4%). For FSH, Device 2b (R^2^ = 0.90) and Device 1 (R^2^ = 0.82) showed strong linearity but had relatively high deviation from linearity (25-35%). Linearity for E_1_3G varied dramatically across devices, with Devices 2a and 2b showing strong linearity and Devices 3 and 4 demonstrating essentially no linear relationship between expected and measured E_1_3G concentrations; the slope was closest to 1 for Device 1 (0.94) and Device 2a had the lowest allowable deviation (12.7). For PDG, two devices showed stronger linearity, Device 1 (R^2^ = 0.93) and Device 2a (R^2^ = 0.90) than the others. These two devices also showed be slopes closest to 1 and maximum allowable deviation was lowest for Device 2a.

**Figure 2:**
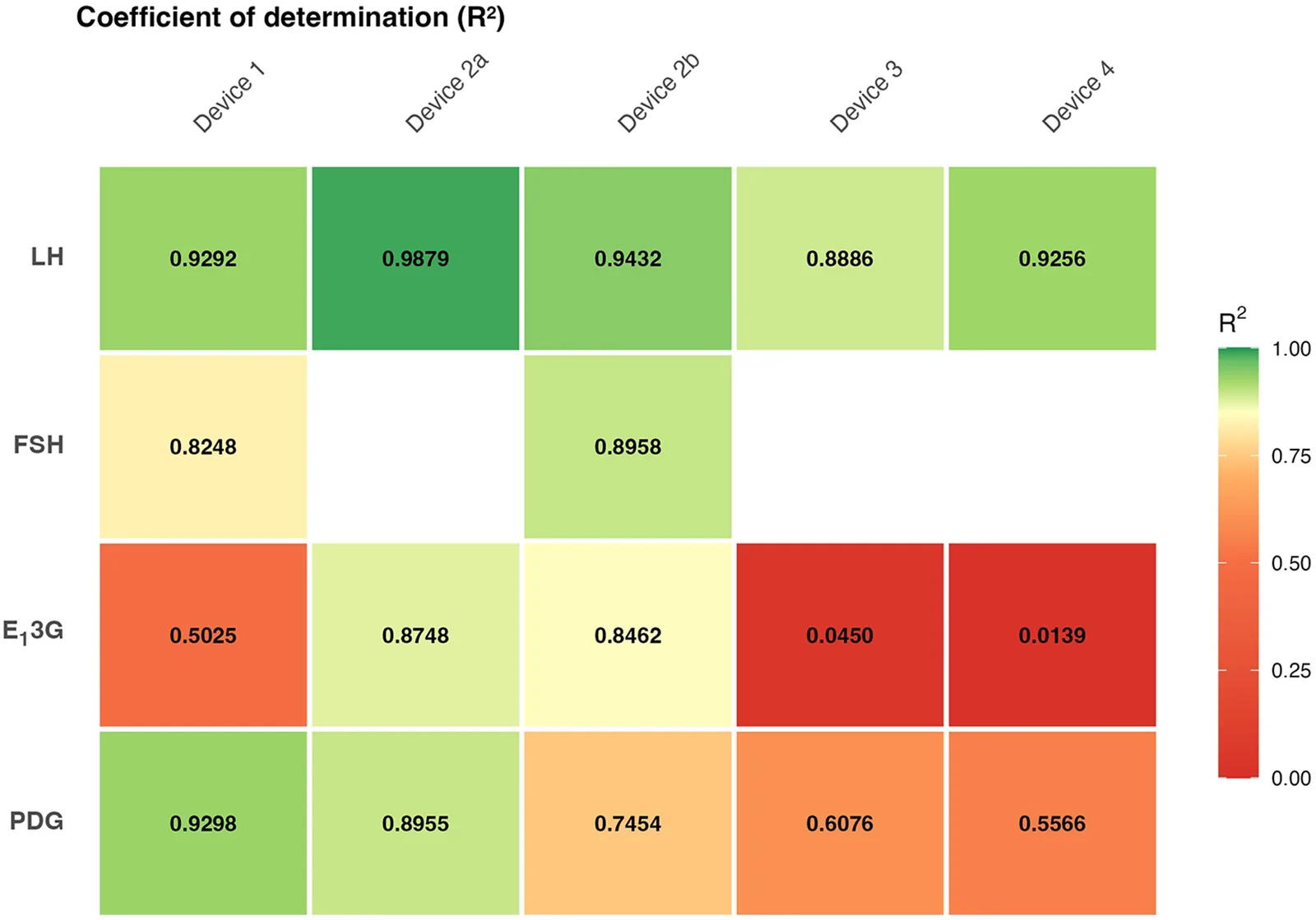
Heatmap of linearity (coefficient of determination = R^2^) across devices and hormones. The colour scale shows higher coefficients of determination (R^2^) as green (high degree of linearity) to red (lowest degree of linearity). Studies for LH, FSH, E_1_3G, and PDG across the quantitative devices (10 replicates per level). Higher values indicate stronger proportional agreement with expected concentrations. This was based on device-reported concentrations with expected calibrator levels (LH/FSH: 5–80 mIU/mL; E_1_3G: 80–400 ng/mL; PDG: 2–24 μg/mL, 10 replicates per level). E_1_3G, estrone-3-glucuronide; FSH, follicle stimulating hormone; LH, luteinizing hormone; PDG, pregnanediol glucuronide.

**Table 2:** Comparison of deviation from linearity statistics.

Analyte	Device	n	Slope (95% CI)	Intercept (95% CI)	R^2^	Max ADL (%)
LH	Device 1	48	0.26 (0.24, 0.28)	-1.11 (-1.99, -0.22)	0.93	72.3
	Device 2a	50	1.00 (0.97, 1.03)	2.54 (1.22, 3.87)	0.99	8.4
	Device 2b	50	1.80 (1.67, 1.93)	-7.76 (-13.05, -2.48)	0.94	373.6
	Device 3	50	1.72 (1.54, 1.89)	-0.22 (-7.50, 7.07)	0.89	61.8
	Device 4	50	0.48 (0.44, 0.52)	-4.14 (-5.78, -2.51)	0.93	64.1
FSH	Device 1	48	0.21 (0.18, 0.24)	-0.06 (-1.25, 1.15)	0.82	25.5
	Device 2b	50	0.71 (0.64, 0.78)	4.76 (1.85, 7.68)	0.90	34.5
E_(1)_3G	Device 1	48	0.94 (0.67, 1.22)	285.70 (210.88, 360.52)	0.50	38.4
	Device 2a	50	0.73 (0.65, 0.81)	94.19 (72.82, 115.55)	0.87	12.7
	Device 2b	50	0.73 (0.64, 0.82)	20.05 (-3.97, 44.06)	0.85	37.0
	Device 3	50	0.20 (-0.07, 0.47)	195.40 (123.66, 267.14)	0.04	50.2
	Device 4	50	0.07 (-0.10, 0.25)	93.99 (46.92, 141.06)	0.01	50.5
PDG	Device 1	48	1.10 (1.01, 1.19)	-0.28 (-1.52, 0.95)	0.93	20.5
	Device 2a	50	0.92 (0.83, 1.01)	0.34 (-0.90, 1.57)	0.90	6.9
	Device 2b	50	0.85 (0.70, 0.99)	-1.27 (-3.21, 0.68)	0.75	154.6
	Device 3	50	0.64 (0.49, 0.79)	11.76 (9.73, 13.79)	0.61	26.4
	Device 4	50	0.48 (0.35, 0.60)	6.95 (5.28, 8.62)	0.56	23.0

CI, confidence interval; Max ADL, maximum allowable deviation from linearity, i.e. maximum percent deviation between the mean measured value and the linear best-fit value at each concentration level.

### 3.3 Limit of Blank and Limit of Detection distributions

Dot plots in **Figure 3** demonstrate deviations from blank for each device-analyte combination. For LH, three devices (Devices 1, 3 and 4) demonstrated near-zero LoB values (0.00–0.10 mIU/mL), indicating consistent baseline readings with minimal noise when measuring blank samples. Device 2a and 2b showed elevated LoB values (3.11 and 0.69 mIU/mL respectively), with Tukey-adjusted pairwise comparisons confirming significant differences from the lower-LoB devices. For FSH (measured only with Devices 1 and 2b), there were significantly different blank and low-concentration measurements between them but not to a degree that would be clinically relevant.

**Figure 3:**
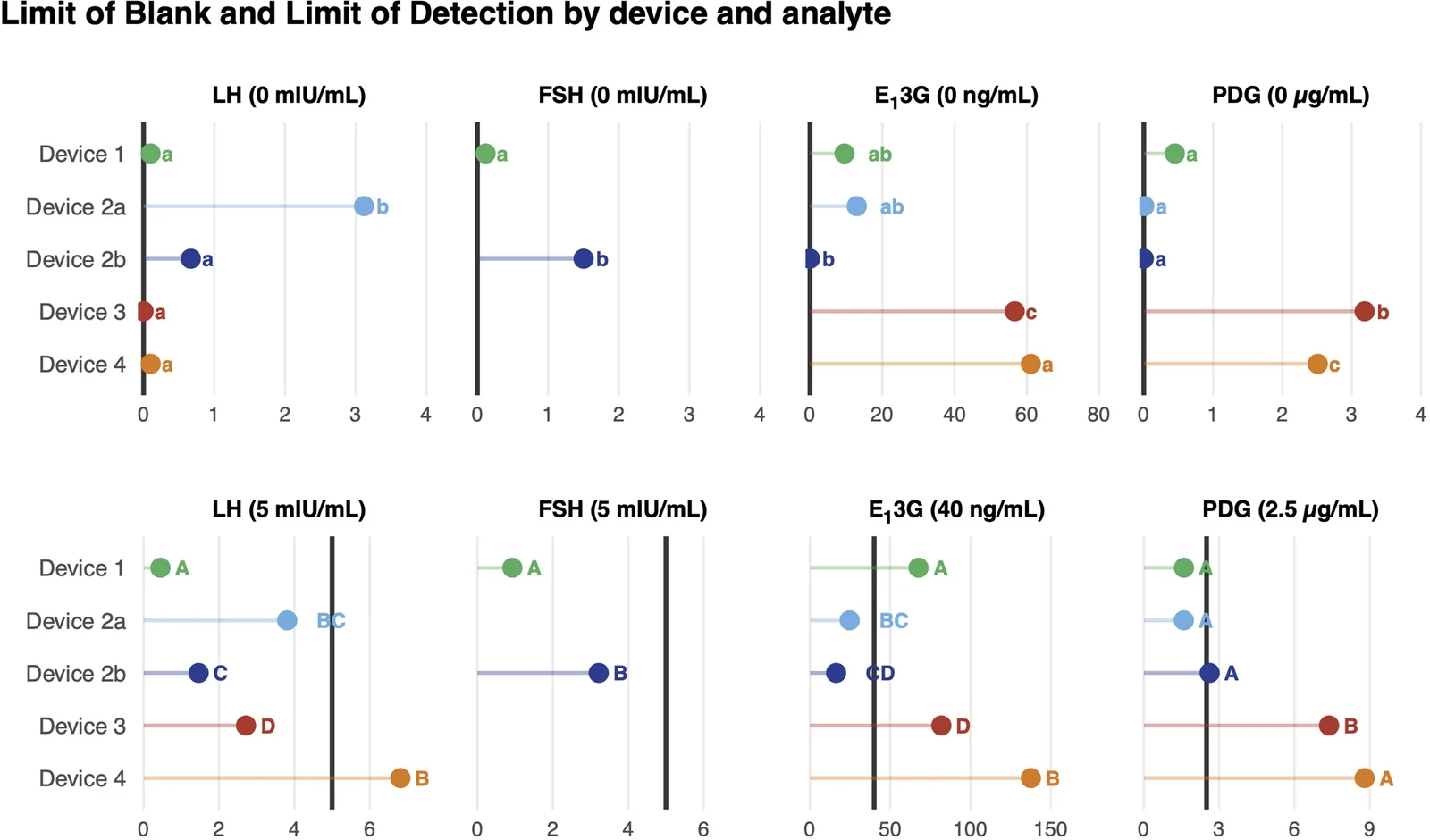
Limit of Blank (LoB) and Limit of Detection (LoD) dot plots. For LoB, the spiked urine target is a concentration of 0 for each hormone, and for LoD, the spiked urine target is 5 mIU/mL for LH and FSH, 40 ng/mL for E_1_3G and 2.5 ug/mL for PDG. Each device/hormone pair was tested 20 times in the artificial urine. Letters in parentheses indicate compact letter displays from Tukey-adjusted pairwise comparisons within each analyte, shown for descriptive purposes and not meant for formal device-level inferential comparisons. A shared letter indicates a similar (not significantly different) pattern between devices. E_1_3G, estrone-3-glucuronide; FSH, follicle stimulating hormone; LH, luteinizing hormone; PDG, pregnanediol glucuronide.

For E_1_3G, Devices 1, 2a and 2b had near-zero LoB values, with minimal noise in measuring blank samples; in contrast with the other devices which had consistently higher readings in blank samples. The lowest E_1_3G LoDs were observed for the same device with the highest linearity (Device 2a/2b). Interpreted against the 50 ng/mL clinical threshold, two devices achieve LoDs near or just below the level of the E_1_3G measurements become clinically meaningful, while the remaining devices have LoDs above the threshold (Devices 1, 3, 4). For PDG, Devices 1 and 2 showed minimal noise for LoB, while the others showed progressively higher LoBs and a similar pattern was found for LoDs (**Figure 3**).

### 3.4 Effect of ambient light

Ambient lighting effects are shown in the heatmap in **Figure 4**. Device 3 showed preserved performance in different lighting conditions, with significant deviations confined to LH at low concentrations (**Figure 4**, upper row). Device 4 was more affected by light: LH readings deviated significantly under both bright and dark conditions at 12.5 and 25 mIU/mL, and PDG readings showed pronounced light-dependent variability at lower concentrations (**Figure 4**, lower row). Effects were generally most pronounced at lower analyte concentrations, suggesting ambient lighting differentially influences the optical analysis of fainter test bands.

**Figure 4:**
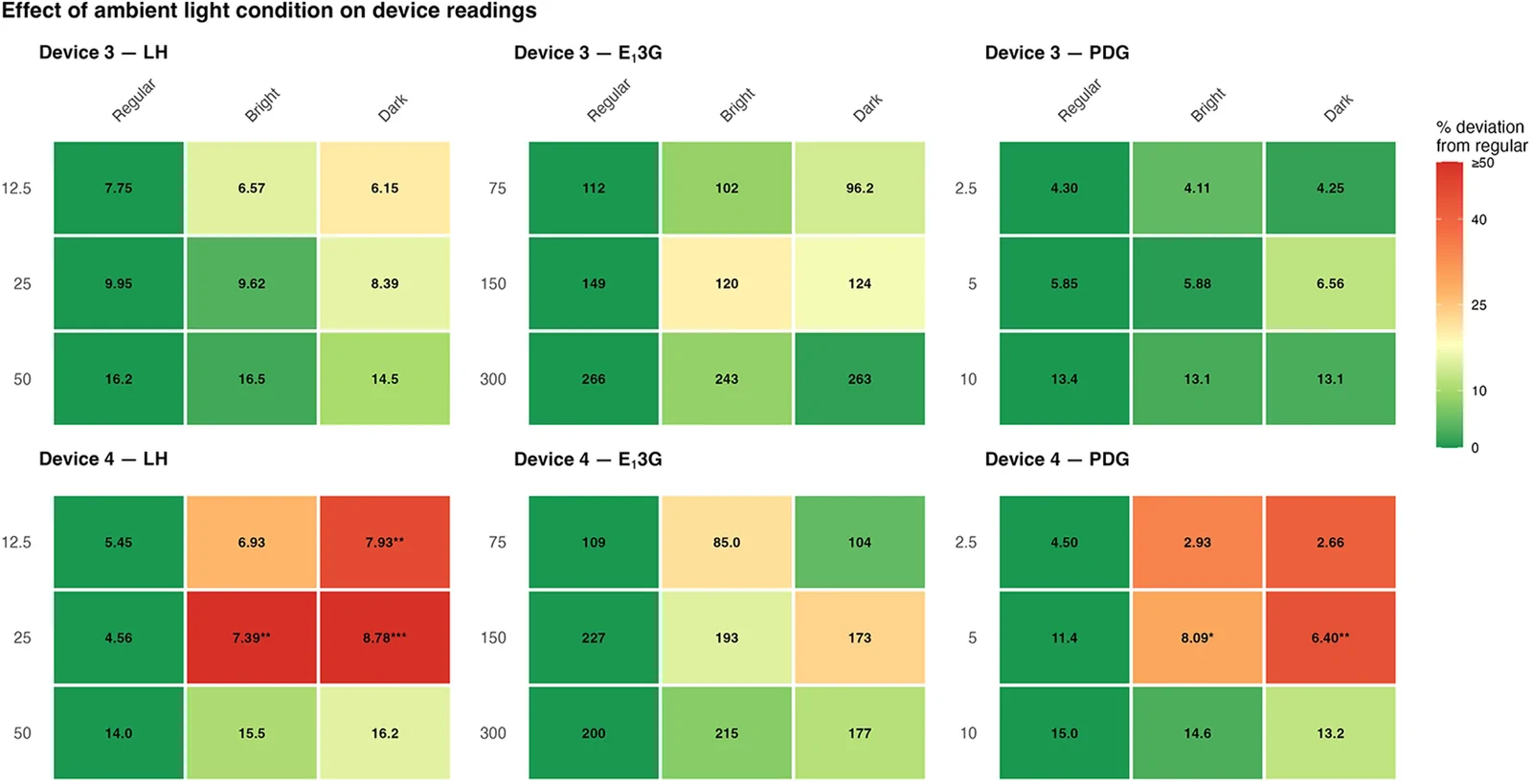
Hormone concentrations in different lighting conditions. Regular refers to ambient room lighting, Bright = 300 lux lighting, Dark = 50 lux lighting. The heatmap colour scale is relative to ambient (regular) light as dark green, with red being a ≥ 50% deviation from the regular light reading. Asterisks on bright and dark cells indicate Dunnett-adjusted p-values vs regular (*p<0.05, **p<0.01, ***p<0.001). Ten replicates per concentration were sequentially tested in the different conditions. Target analyte concentrations are to the left of each heatmap panel. E_1_3G, estrone-3-glucuronide; FSH, follicle stimulating hormone; LH, luteinizing hormone; PDG, pregnanediol glucuronide.

### 3.5 Specificity analysis

Specificity was evaluated for each device by challenging assay channels with non-target hormones at specified concentrations: human chorionic gonadotropin (hCG, 100 mIU/mL), thyroid-stimulating hormone (TSH, 0.025 mIU/mL), 17α-hydroxyprogesterone (17α-OHP, 5 µg/mL), pregnenolone (5 µg/mL), cortisol (5 µg/mL), and progesterone (5 µg/mL) (**Figure 5**).

**Figure 5:**
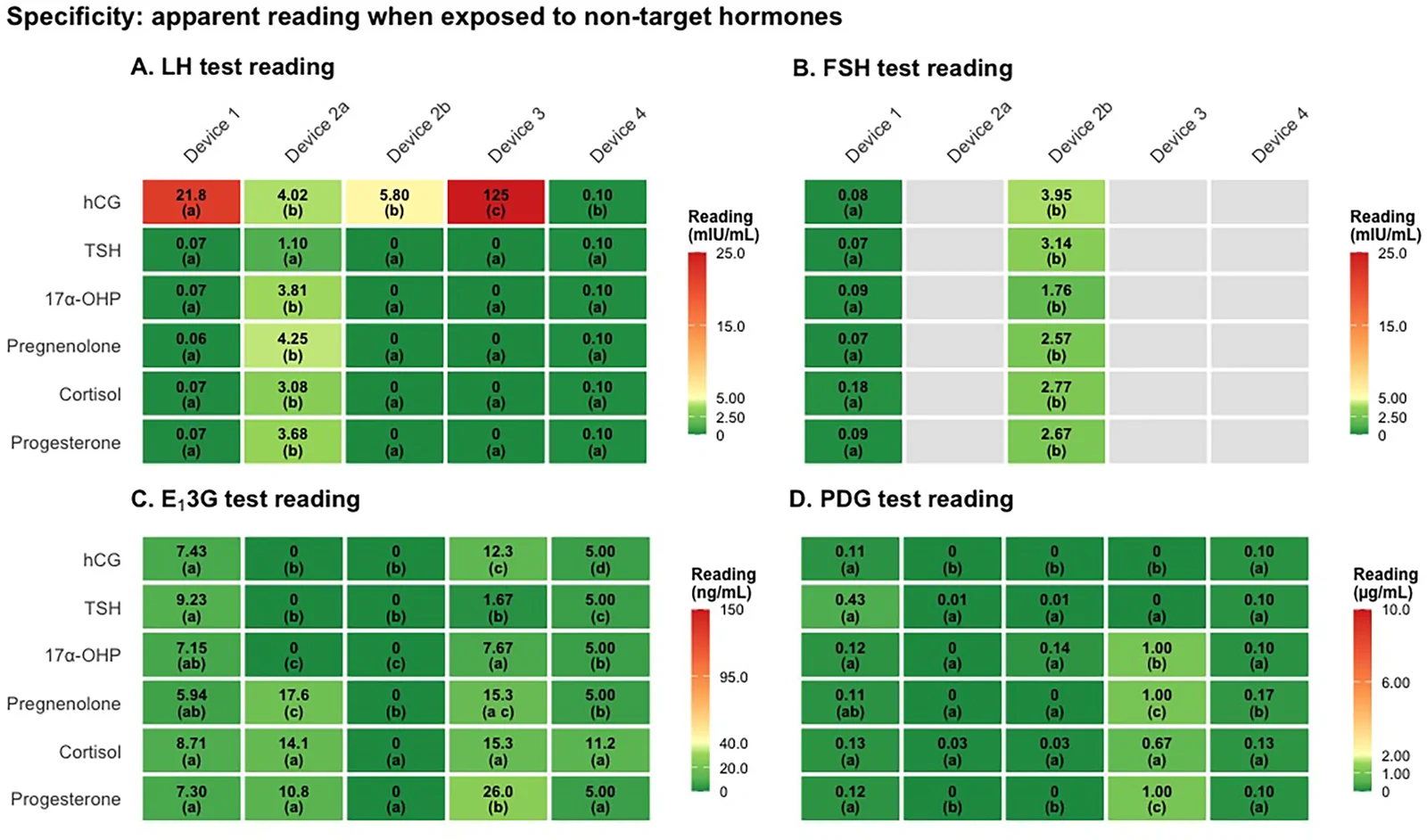
Heatmap of each device’s hormone reading in the presence of each interferent in artificial urine. Interferents are labeled to the left of the heatmap. The colour scale reflects clinical false-positive risk relative to the clinical decision threshold for each analyte; readings below threshold but above zero represent cross-reactivity that may not produce clinical false positives but indicate analytical specificity limitations. Letters in parentheses indicate compact letter displays from Tukey-adjusted pairwise comparisons within each analyte. A shared letter indicates a similar (not significantly different) pattern between devices. 3 replicates per concentration were sequentially tested with each device-analyte combination. 17-α-OHP, 17-α-hydroxyprogesterone, E_1_3G, estrone-3-glucuronide; FSH, follicle stimulating hormone; LH, luteinizing hormone; PDG, pregnanediol glucuronide.

The most pronounced cross-reactivity occurred with LH testing when challenged with hCG, reflecting the well-established structural homology between the alpha subunits of LH and hCG. All devices except one had (Device 4) significant LH-hCG cross-reactivity. Device 4 measuring the beta-LH subunit avoided an LH false-positive entirely. For all other interferent–target combinations, cross-reactivity was minimal and clinically inconsequential.

## 4 Discussion

This study presents a comprehensive analytical framework to compare commercially available at-home fertility monitoring devices. The pre-registered analysis plan was meant to minimize bias given that the data was provided by one of the device manufacturers (Quanovate Tech Inc. who developed Device 2), but also to provide an opportunity for replicability using this analytic framework to further the validation trajectory for these devices. Analytical performance varied significantly among devices. Some demonstrated high precision but lower accuracy, resulting in systematical biased at spiked concentrations—a pattern that may reflect manufacturer-specific calibration choices rather than measurement error. Others showed strong linearity but higher LoB and LoD. The devices that are strip-based and dependent on smartphone imaging may be vulnerable to bias in low lighting conditions.

All four devices had excellent LH linearity (**Figure 2**), consistent with LH being one of the most established urinary hormone analytes, with decades of assay development (since the 1980s) (33) and widespread use in home testing. Accuracy, precision and linearity are all important in considering which device to use in identifying the beginning of the fertile window, with the primary hormone of interest being E_1_3G.

The limit of blank (LoB) and limit of detection (LoD) findings (**Figure 3**) provide context for interpreting how reliably each device distinguishes signal from noise at the lower end of its evaluated operating range. The lowest concentrations in our linearity assessments — 5 mIU/mL for LH and FSH, 40 ng/mL for E_1_3G, and 2.5 µg/mL for PDG — represent the lower end of the tested range for each analyte. Device performance at these concentrations reflects analytical behaviour under controlled artificial-urine conditions and should not be interpreted as evidence of clinical performance or as defining practical floors for clinical use. Low end imprecision is not surprising for immunoassays where signal-to-noise deteriorates near the lower bound of the measured range. This noise at blank or low concentrations is mitigated as the hormone rises above its baseline and signal-to-noise improves. Therefore, the noise represented below the limit of detection was less concerning for all four devices, but for E_1_3G and PDG, two devices did not reliably discriminate at the lower end of their evaluated operating range.

For those devices that require taking a picture of a test strip, the impact of bright or dark lighting conditions on a test result is important to consider when providing instructions to users on optimal lighting and timing for taking photos of the test strips (34). While not specifically tested in this study, another important consideration is how much time a test strip is left to develop before a picture is taken of the result, since the intensity of the line can change based on the time elapsed (35) as well as environmental conditions like lighting. Timed testing helps to control one aspect of this variability.

Minimizing the risk of interference from other hormones potentially present in a sample is also a key consideration of analytic validity in immunoassays (20). The most relevant finding with respect to interference was the cross-reactivity of LH and hCG. LH and hCG share an identical α-subunit and approximately 85% β-subunit sequence identity, the biochemical basis for their cross-reactivity. LH-based ovulation tracking is practically much less likely to be done at the end of a cycle, nevertheless it may have implications in women with inconsistent ovulation or long cycles. The contrast between hCG cross-reactivity and the near-zero responses to TSH (which shares the α-subunit but only 40-45% β-subunit homology with LH) suggests that the immunoassays appropriately target β-subunit epitopes, and that hCG cross-reactivity reflects the unique LH-hCG structural similarity rather than general specificity failure. For all other interferents tested — including the steroid hormones cortisol, pregnenolone, 17α-hydroxyprogesterone, and progesterone — cross-reactivity was clinically inconsequential across all device-analyte combinations. Although there were some elevated levels for some devices, these were well below the limit of detection for these hormones in a range that would not be interpreted as elevated levels of those hormones. In the case of Device 2a showing mildly elevated levels for LH across all interferents, this is likely artefact at the device’s concentration floor, given that LoB is also noisier for LH in the same device.

### 4.1 Strengths and limitations

The main strength of this study was establishing a pre-registered analysis plan applied to commercially available quantitative at-home urine monitors currently in North America. Artificial urine with hormones at specified concentrations was used as a standard to ensure a consistent matrix was used for each device and analyte under the conditions specified by each manufacturer.

With 10 replicates per analyte concentration level, this study was adequately powered to detect significant differences between the device-analyte combinations for precision and accuracy, but larger sample sizes would be appropriate to detect more subtle differences between the different validity measures used here. These experiments were informed by CLSI standards and are preliminary, but to be fully compliant, this would require reporting between-day, between-run, lot-to-lot and device-to-device comparisons. An important concept to consider when using replicates from a single instrument or lot is pseudoreplication, where repeating very similar test environments (i.e. same device and lot) does not reflect the heterogeneity needed to assess the test more broadly. Using multiple lots reduces but does not eliminate pseudoreplication, therefore, comparisons between these devices should be interpreted as descriptive under the tested conditions rather than as evidence of population-level device differences.

Additional interferents and higher concentrations of interferents should be tested in future studies. For example, TSH was tested at a relatively low concentrations, and should be evaluated in future studies at more clinical meaningful concentrations. However, it should be noted that TSH is not usually in the urine but were tested in an artificial urine matrix. Similarly, progesterone, 17-OHP and pregnenolone as free steroids are also not found in human urine because they are glucuronidated by the liver for urinary excretion. Other important factors not fully reproduced in artificial urine include pH, salt and proteins, hydration status (specific gravity). Thus, this analytic framework requires testing in human urine to further explore the analytic reliability of these testing systems in a clinical environment sine device difference here may reflect the artificial matrix and manufacturer-specific calibration rather than clinical differences. Deeper interference testing (including common medications and supplements) at realistic concentrations should also be tested in future studies since oral medications and supplements may bias device outputs (e.g. biotin supplementation). The current analysis plan could be replicated by more manufacturers or other research groups to corroborate and expand on these findings. In addition to the use of artificial urine samples, human urine samples will also be important to include in future validity experiments.

The variable analytic performance for the conjugated metabolites of estrogen and progesterone relates to the combination of their physiologic variability along with the challenges related to the assays themselves (36). Since these devices are on a development trajectory, their assays and monitoring tools are likely to improve with time. Commercial bias is a significant component of any device comparison, and we acknowledge that the data analyzed here were provided by only one manufacturer, which may reduce reproducibility. Nevertheless, this analysis is the first step in moving towards a reproducible analysis plan to assess these and other devices as they evolve. Since monitor and test strip development is a dynamic process, a single study like this one should not be seen as the final word on monitor validation but as an iterative process that promotes open science practices among manufacturers. Analytical performance of these devices could change relative to each other and the current study’s results need to be contextualized to each device and test stick model (serial and lot numbers are included in the Appendix). Independent replication by other laboratories using multiple instruments, lots and first-morning human urine samples will be required to establish generalizable performance characteristics.

## 5 Conclusions

This study establishes a foundational analytical performance framework that can be applied in future research on menstrual-cycle hormone monitoring. The findings in this study are limited to analytical behaviour under controlled artificial-urine conditions and do not establish clinical validity or commutability in real human urine. Independent replication and clinical studies with appropriate ovulation reference standards are required before these results can inform clinical decision-making or device selection.

## Data Availability

The raw data supporting the conclusions of this article will be made available by the authors, without undue reservation with appropriate ethics approval.

## Appendix: device models and lot numbers

**Device 1**

1. Device model: Inito Fertility Monitor
2. Strip version: Inito Fertility Monitor Test Strips
3. Lot numbers: Inito Fertility Monitor(D03HA1FG and 6461C2F0), Inito Fertility Monitor Test Strips(7GJ570Q0, 2F4EP6Q0, and5YWD30Q0)
4. Software version: 5.40.
5. Smartphone model: iPhone 14 Plus

**Device 2a/2b**

1. Device model : Mira Analyzer
2. Strip version: Mira Fertility Max Wand (2a) (Fluorescence Immunoassay) and Mira Ultra4 Wand (2b) (Fluorescence Immunoassay)
3. Lot numbers: Mira Analyzer(E3605AC3D6), Mira Fertility Max Wand (2025031204) and Mira Ultra4 Wand (2025021801)
4. Software and firmware versions: V3.5.11.5 and 01.07.01.31.
5. Smartphone models: not required

**Device 3**

1. Device model: Oova Testing System
2. Strip version: Oova Test Strips
3. Lot numbers: EFP01 and OFP02
4. Software: Oova 3.022
4. Smartphone model: iPhone 7/11/15

**Device 4**

1. Device model: Proov Testing System
2. Strip Version: Proov Test Strips
3. Lot numbers: LN1744
4. Software: Proov 3.1.4
5. Smartphone model: iPhone 7/11/15

## References

[1] Yong PJ Khan Z Wahl K Bouchard TP Doyle-Baker PK Prior JC . Menstrual health literacy, equity and research priorities. J Obstet Gynaecol Can. (2024) 46:102–4. doi: 10.1016/j.jogc.2024.102711 39701613

[2] Hart RJ D’Hooghe T Dancet EAF Aurell R Lunenfeld B Orvieto R . Self-monitoring of urinary hormones in combination with telemedicine — a timely review and opinion piece in medically assisted reproduction. Reprod Sci. (2022) 29:3147–60. doi: 10.1007/s43032-021-00754-5 34780023 PMC8592080

[3] Brezina PR Haberl E Wallach E . At home testing: optimizing management for the infertility physician. Fertility Sterility. (2011) 95:1867–78. doi: 10.1016/j.fertnstert.2011.01.001 21324446

[4] Kassam A Overstreet JW Snow-Harter C Souza MJD Gold EB Lasley BL . Identification of anovulation and transient luteal function using a urinary pregnanediol-3-glucuronide ratio algorithm. Environ Health Perspect. (1996) 104:408–13. doi: 10.1289/ehp.96104408 8732951 PMC1469335

[5] Hambridge HL Mumford SL Mattison DR Ye A Pollack AZ Bloom MS . The influence of sporadic anovulation on hormone levels in ovulatory cycles. Hum Reprod. (2013) 28:1687–94. doi: 10.1093/humrep/det090 23589536 PMC3657125

[6] Lynch KE Mumford SL Schliep KC Whitcomb BW Zarek SM Pollack AZ . Assessment of anovulation in eumenorrheic women: comparison of ovulation detection algorithms. Fertility Sterility. (2014) 102:511–518.e2. doi: 10.1016/j.fertnstert.2014.04.035 24875398 PMC4119548

[7] Robinson JE Ellis JE . Mistiming of intercourse as a primary cause of failure to conceive results of a survey on use of a home-use fertility monitor. Curr Med Res Opin. (2007) 23:301–6. doi: 10.1185/030079906x162863 17288684

[8] Wilcox AJ Weinberg CR Baird DD . Timing of sexual intercourse in relation to ovulation — effects on the probability of conception, survival of the pregnancy, and sex of the baby. N Engl J Med. (1995) 333:1517–21. doi: 10.1056/nejm199512073332301 7477165

[9] Alizzi FJ Showman HAK Fawzi HA Iraq, Department of Obstetrics, Gynecology and Infertility, College of Medicine, Al Mustansiriyah 1 University, Baghdad . Predictors for response to letrozole as an ovulation induction in anovulatory infertile polycystic ovarian syndrome women. Iraqi J Embryos Infertility Res. (2019) 9:89–110. doi: 10.28969/ijeir.v9.i1.r6

[10] Wegrzynowicz AK Beckley A Eyvazzadeh A Levy G Park J Klein J . Complete cycle mapping using a quantitative at-home hormone monitoring system in prediction of fertile days, confirmation of ovulation, and screening for ovulation issues preventing conception. Medicina. (2022) 58:1853. doi: 10.3390/medicina58121853 36557055 PMC9783738

[11] Bouchard TP Fehring R Meyers M Stujenske TM Boychuk L Smith A . Modeling fertile window differences across the reproductive lifespan with quantitative urine hormone monitoring of the menstrual cycle. J Ovarian Res. (2026). doi: 10.1186/s13048-026-02184-w 42443950 PMC13660213

[12] Gimunová M Paulínyová A Bernaciková M Paludo AC . The prevalence of menstrual cycle disorders in female athletes from different sports disciplines: a rapid review. IJERPH. (2022) 19:14243. doi: 10.3390/ijerph192114243 36361122 PMC9658102

[13] Bouchard TP Fehring RJ Schneider MM . Achieving pregnancy using primary care interventions to identify the fertile window. Front Med. (2018) 4:250. doi: 10.3389/fmed.2017.00250 29376054 PMC5767237

[14] Carson SA Kallen AN . Diagnosis and management of infertility: a review. JAMA. (2021) 326:65. doi: 10.1001/jama.2021.4788 34228062 PMC9302705

[15] Ecochard R Boehringer H Rabilloud M Marret H . Chronological aspects of ultrasonic, hormonal, and other indirect indices of ovulation. BJOG: Int J Obstetrics Gynaecology. (2001) 108:822–9. doi: 10.1111/j.1471-0528.2001.00194.x 11510707

[16] Njagi P Groot W Arsenijevic J Dyer S Mburu G Kiarie J . Financial costs of assisted reproductive technology for patients in low- and middle-income countries: a systematic review. Hum Reprod Open. (2023) 2023:hoad007. doi: 10.1093/hropen/hoad007 36959890 PMC10029849

[17] Brodeur TY Grow D Esfandiari N . Access to fertility care in geographically underserved populations, a second look. Reprod Sci. (2022) 29:1983–7. doi: 10.1007/s43032-022-00991-2 35680726 PMC9181892

[18] Johnson S Bond S Grace B Marriott L . Increased chance of live birth following use of connected ovulation test system: outcome results from a randomized controlled trial. Women’s Health Rep. (2022) 3:60–6. doi: 10.1089/whr.2021.0102 35136878 PMC8812496

[19] Cromack SC Walter JR . Consumer wearables and personal devices for tracking the fertile window. Am J Obstetrics Gynecology. (2024) 231:516–23. doi: 10.1016/j.ajog.2024.05.028 38768799

[20] Sturgeon CM Viljoen A . Analytical error and interference in immunoassay: minimizing risk. Ann Clin Biochem. (2011) 48:418–32. doi: 10.1258/acb.2011.011073 21750113

[21] Newman MS Curran DA Mayfield BP Saltiel D Stanczyk FZ . Assessment of estrogen exposure from transdermal estradiol gel therapy with a dried urine assay. Steroids. (2022) 184:109038. doi: 10.1016/j.steroids.2022.109038 35483542

[22] Glynne S Reisel D Kamal A Neville A McColl L Lewis R . The range and variation in serum estradiol concentration in perimenopausal and postmenopausal women treated with transdermal estradiol in a real-world setting: a cross-sectional study. Menopause. (2025) 32:103–11. doi: 10.1097/GME.0000000000002459 39689249 PMC12147738

[23] Pattnaik S Das D Venkatesan VA Rai A . Predicting serum hormone concentration by estimation of urinary hormones through a home-use device. Hum Reprod Open. (2022) 2023:hoac058. doi: 10.1093/hropen/hoac058 36654688 PMC9838318

[24] Bouchard TP Bedaiwy M Talhouk A Nakhuda G Yong PJ Doyle-Baker PK . Validating ovulation prediction and confirmation with the Mira monitor: blinded ultrasound and serum hormone comparison. Reprod BioMedicine Online. (2026) 53(5):105881. doi: 10.1016/j.rbmo.2026.105881 42600546

[25] Hills E Woodland MB Divaraniya A . Using hormone data and age to pinpoint cycle day within the menstrual cycle. Medicina. (2023) 59:1348. doi: 10.3390/medicina59071348 37512159 PMC10384168

[26] Bouchard TP Pierson R Ecochard R Lane M Alger G Diment C . Using corpus luteum formation with dominant follicle collapse to improve the criteria for identifying the day of ovulation. Reprod BioMedicine Online. (2026) 53(1). doi: 10.1016/j.rbmo.2026.105660 42105719

[27] Gronowski AM Nerenz RD . Assessing the risk of false negative point-of-care urinary human chorionic gonadotropin device results due to beta core fragment. Clin Biochem. (2015) 48:97–8. doi: 10.1016/j.clinbiochem.2015.01.001 25578769

[28] Bouchard T Yong P Doyle-Baker P . Establishing a gold standard for quantitative menstrual cycle monitoring. Medicina. (2023) 59:1513. doi: 10.3390/medicina59091513 37763632 PMC10533176

[29] Bouchard TP Yong PJ Doyle-Baker PK . Analytical validation and comparison of four quantitative urinary hormone monitoring systems for fertility tracking [Analysis Plan. In: Osf Registries. Washington, DS.: Center for Open Science. (2026). Available online at: https://osf.io/w8eh5/ (Accessed April 2026).

[30] Clinical and Laboratory Standards Institute . Evaluation of precision of quantitative measurement procedures; approved guideline—third edition. Wayne, PA: Clinical and Laboratory Standards Institute (CLSI). (2014). Available online at: https://clsi.org/standards/products/method-evaluation/documents/ep05/ (Accessed May 28, 2026).

[31] European Medicines Agency . ICH guideline M10 on bioanalytical method validation [Guideline. In: . Amsterdam (2022). Available online at: https://www.ema.europa.eu/en/ich-m10-bioanalytical-method-validation-scientific-guideline (Accessed May 28, 2026).

[32] Clinical and Laboratory Standards Institute . Evaluation of detection capability for clinical laboratory measurement procedures; approved guideline—second edition. In: . Wayne, PA (2012). Available online at: https://clsi.org/standards/products/method-evaluation/documents/ep17/ (Accessed May 28, 2026).

[33] Knee GR Feinman MA Strauss JF Blasco L Goodman DBP . Detection of the ovulatory luteinizing hormone (LH) surge with a semiquantitative urinary LH assay. Fertility Sterility. (1985) 44:707–9. doi: 10.1016/S0015-0282(16)48993-4 4054353

[34] Foysal KH Seo SE Kim MJ Kwon OS Chong JW . Analyte quantity detection from lateral flow assay using a smartphone. Sensors. (2019) 19:4812. doi: 10.3390/s19214812 31694281 PMC6864604

[35] Colombo M Bezinge L Rocha Tapia A Shih CJ De Mello AJ Richards DA . Real-time, smartphone-based processing of lateral flow assays for early failure detection and rapid testing workflows. Sens Diagn. (2023) 2:100–10. doi: 10.1039/D2SD00197G 36741250 PMC9850356

[36] Newman M Pratt SM Curran DA Stanczyk FZ . Evaluating urinary estrogen and progesterone metabolites using dried filter paper samples and gas chromatography with tandem mass spectrometry (GC–MS/MS). BMC Chem. (2019) 13:20. doi: 10.1186/s13065-019-0539-1 31384769 PMC6661742

